# Embolization of pseudoaneurysms in the ureteral branch of the renal artery

**DOI:** 10.1186/s42155-023-00400-y

**Published:** 2023-10-23

**Authors:** Atsushi Saiga, Takeshi Aramaki, Rui Sato, Kazuhisa Asahara, Hironori Goto

**Affiliations:** 1 Division of Interventional Radiology, Shizuoka Cancer Center, 1007 Shimonagakubo, Nagaizumi-Cho, Shizuoka, Sunto-Gun 411-8777 Japan; 2 Division of Diagnostic Radiology, Shizuoka Cancer Center, 1007 Shimonagakubo, Nagaizumi-Cho, Shizuoka, Sunto-Gun 411-8777 Japan

**Keywords:** Ureteral branch artery embolization, Pseudoaneurysm, Ureteral involvement, Gastric cancer

## Abstract

**Background:**

Although transcatheter arterial embolization for pseudoaneurysms is already well-established, ureteral artery pseudoaneurysm embolization is extremely rare. The present case shows a successful transcatheter arterial embolization for pseudoaneurysms in the ureteral branch of the renal artery due to ureteral invasion from gastric cancer.

**Case presentation:**

A 57-year-old female presented with gross hematuria after treatments for poorly differentiated gastric adenocarcinoma. A contrast-enhanced computed tomography revealed pseudoaneurysms around the right ureter with a massive hematoma in the right ureter and bladder. The diagnosis was ureteral branch pseudoaneurysms resulting from possible retroperitoneal invasion due to pelvic lymph node metastasis of gastric cancer. Transcatheter arterial embolization was performed using gelatin particles, successfully controlling her hematuria without complications.

**Conclusions:**

Ureteral branch artery embolization, although extremely rare, may be an effective and safe treatment option.

## Background

Urinary tract obstruction is an occasional complication in patients with gastric cancer [1]. Undifferentiated gastric cancer tends to spread and infiltrate along the vessels, nerves, and lymphatic without forming a mass [2]. Infiltration of the ureteral wall weakens the ureteral branch arterial wall, leading to pseudoaneurysm formation. Here, we present a case of ureteral branch pseudoaneurysm caused by possible ureteral invasion from gastric cancer without clear mass formation that was successfully treated by transcatheter arterial embolization (TAE). TAE is a common treatment for pseudoaneurysms, using various embolic materials such as coils, gelatin sponge, cyanoacrylate glue, ethanol sclerosant, and detachable balloons [3]. However, ureteral branch artery embolization has been reported in only one case [4].

## Case presentation

**Fig. 1:**
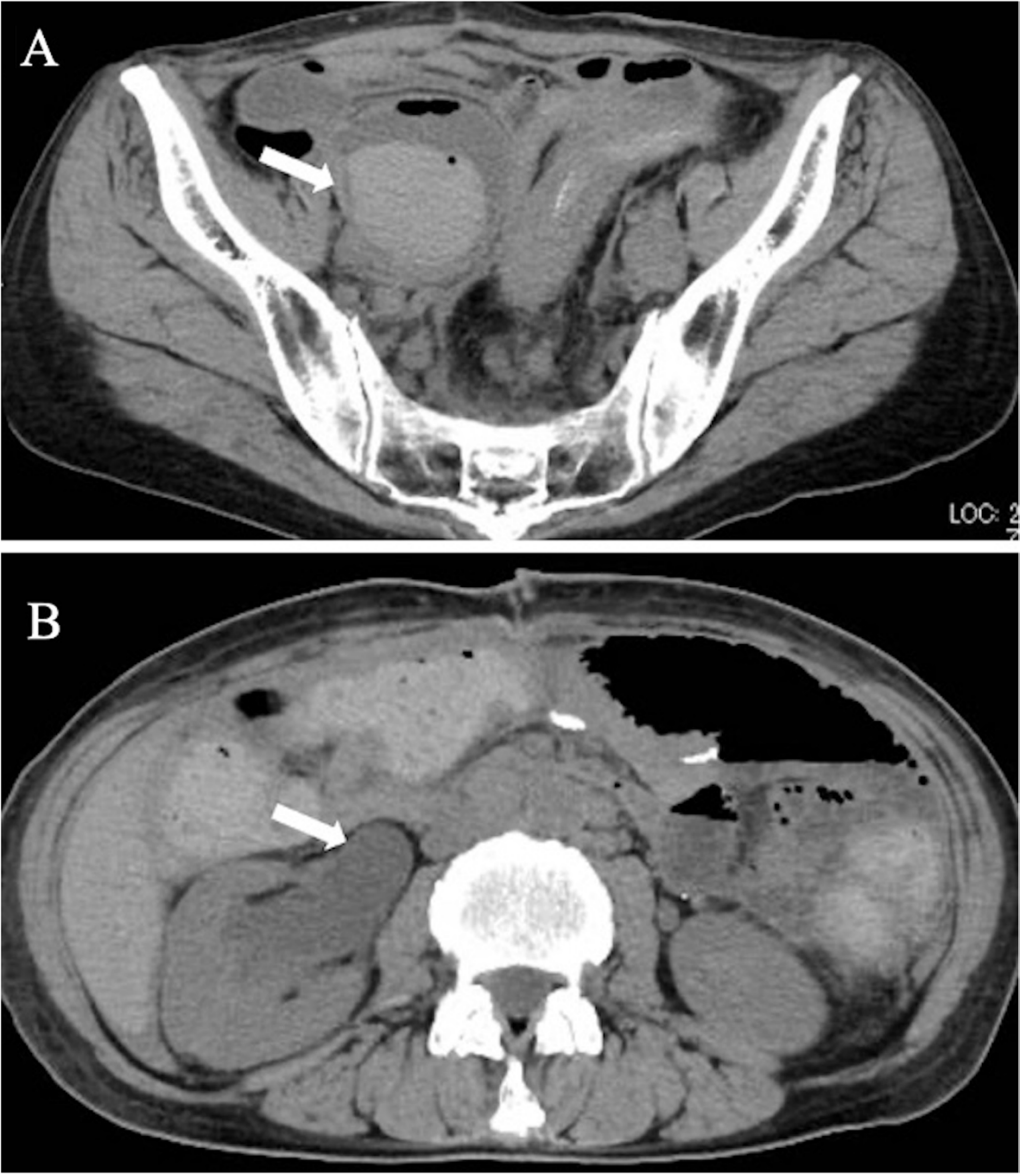
Noncontrast-enhanced computed tomography shows (**A**) a massive hematoma in the right ureter and bladder (arrow) and (**B**) right hydronephrosis (arrow)

**Fig. 2:**
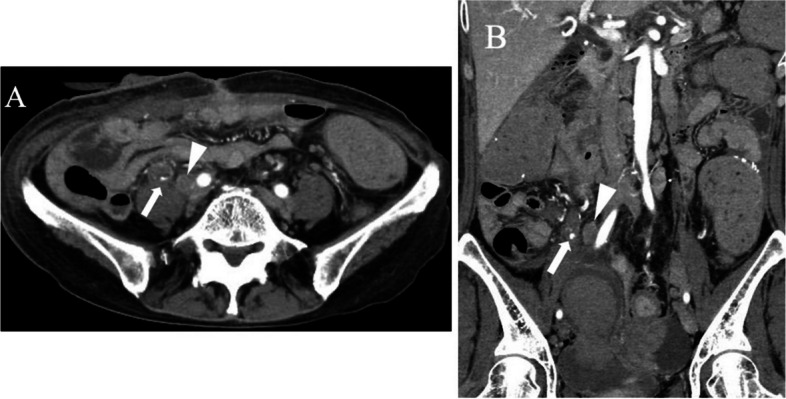
Contrast-enhanced arterial dominant phase demonstrates a pseudoaneurysm around the right ureter (**A**) in the axial and (**B**) coronal images. The arrows in (**A**) and (**B**) indicate the pseudoaneurysm, while the arrowheads in (**A**) and (**B**) show the right common iliac lymph node metastasis

**Fig. 3:**
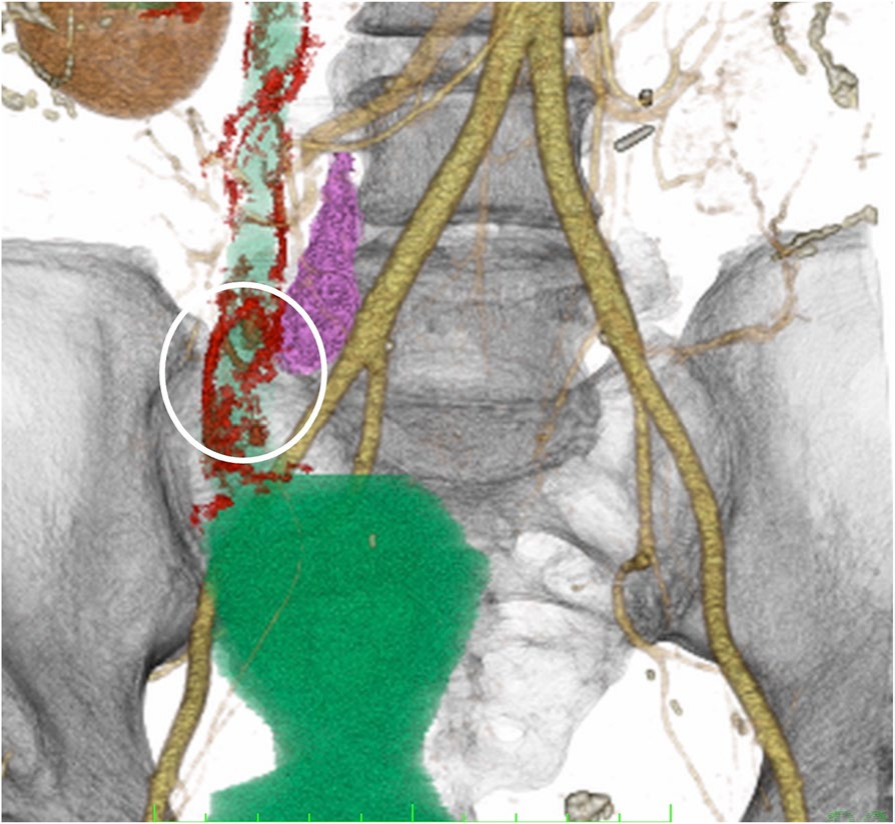
The volume-rendered image from the contrast-enhanced arterial dominant phase gives a three-dimensional overview of the multiple tiny aneurysms (circle), right common iliac lymph node metastasis (purple color), right ureteral branch (red color), and right urethra-bladder anastomosis (green color)

A 57-year-old female presented with gross hematuria. She underwent distal gastrectomy and ovarian resection for gastric cancer and bilateral ovarian metastases 11 years ago, with a pathological diagnosis of poorly differentiated adenocarcinoma. Adjuvant therapy with an oral fluoropyrimidine (S-1) was followed for about 1 year, but sigmoid colon metastasis was subsequently found. Five years prior to the embolization, she underwent sigmoid colon resection and received adjuvant S-1 therapy for one year. Three years after that, due to right extraperitoneal recurrence in the right pelvic wall, she underwent ileocecal resection with concomitant right ureter resection, right urethra-bladder anastomosis, partial rectal resection, and hysterectomy. Adjuvant therapy with S-1 plus docetaxel has been ongoing for 10 months. However, a recent computed tomography (CT) revealed a narrowed sigmoid colon due to peritoneal dissemination. As a result, a colostomy was created, and the patient's hematuria worsened after the operation. Subsequent CT imaging showed a massive hematoma in the right ureter and bladder, along with right hydronephrosis in the noncontrast phase (Fig. 1A and B). Pseudoaneurysms were detected around the right ureter in the arterial dominant phase (Fig. 2A and B) and in the volume-rendered image (Fig. 3).

**Fig. 4:**
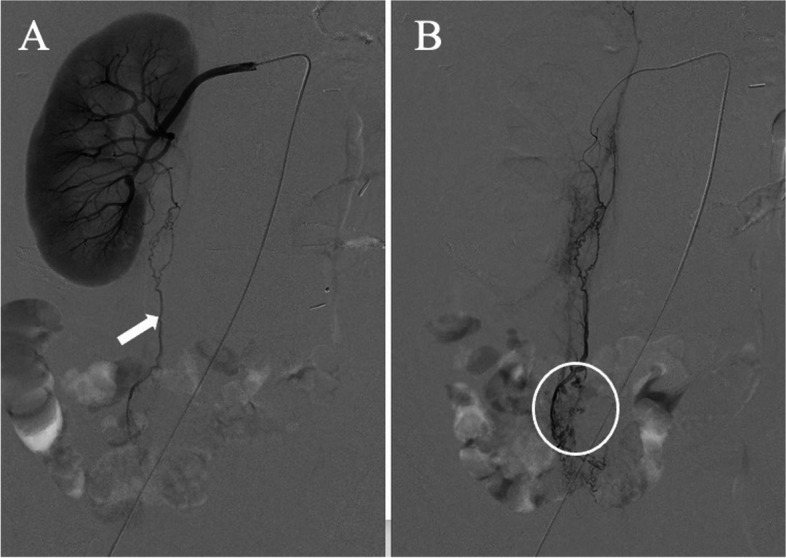
**A** Right renal arteriography shows a ureteral branch (arrow) and (**B**) ureteral branch arteriography depicts multiple tiny aneurysms (circle)

Right femoral artery catheterization was performed under local anesthesia using the Seldinger technique. A 3-Fr diagnostic catheter (Hook type; Medikit; Tokyo, Japan; 65 cm) was inserted through a 3-Fr sheath and placed at the right renal artery. Renal arteriography showed the ureteral branch (Fig. 4A), and a 2.2/2.4-Fr microcatheter (Carnelian Marvel; Tokai Medical; Aichi, Japan; 135 cm) was advanced in the branch. The arteriography demonstrated multiple tiny pseudoaneurysms (Fig. 4B). Gelatin sponge particles were made by pumping it back and forth about 20 times using two syringes attached on a three-way stopcock [5]. The particles with size distribution of mainly < 1 mm [6] were used for the embolization. Postprocedural angiography revealed the disappearance of the pseudoaneurysms. Subsequently, right internal iliac arteriography was performed. Although aneurysms or extravasation were not clearly seen, the iliolumbar artery ran near the abovementioned aneurysms and was embolized using the gelatin sponge particles as well. The patient was discharged without any complications 10 days after the embolization. One month after the embolization, follow-up contrast-enhanced CT indicated no aneurysms or hematoma in the ureter and the bladder. However, her bone metastases and peritoneal dissemination were exacerbated, and her disease condition was uncontrollable. She died 5 weeks after the embolization.

## Discussion

Gastric cancer can spread to the paraaortic area or pelvis, causing ureteral obstruction [7]. Ureteric involvement by metastatic diseases can occur through three different ways: direct invasion of the ureter by a tumor in a neighboring organ, metastasis to lymph nodes around the ureter resulting in retroperitoneal invasion, or distant metastasis [2, 7]. This case was considered to be close to the second scenario as the pseudoaneurysms were found adjacent to a soft-tissue shadow from the right common iliac lymph node metastasis. The retroperitoneal invasion could occur in terms of the trait that the undifferentiated gastric cancer would infiltrate without forming a mass; however, confirming it from the CT images was difficult [2]. However, there are no reports addressing ureteral branch aneurysm due to retroperitoneal invasion, and TAE for the ureteral branch is extremely rare. To the best of our knowledge, there is only one study by Kase et al. [4], who successfully performed embolization using coils and Abiten® for the ureteral branch from the renal artery without complications after an injury caused by cystoscopic removal of a Double-J stent.

The ureteral branches derive from multiple arteries [8]. The upper part receives its blood supply from a branch of the renal artery, while the middle part is supplied by branches from the common iliac arteries, the abdominal aorta, and the gonadal arteries. The most distal part receives blood from branches of the superior and inferior vesicular arteries. The blood supply of the ureter is segmental through these branches, which anastomose on the adventitia covering its wall [9]. Gelatin sponge particles could be safer than others, considering that the distance between the tip of the microcatheter and the pseudoaneurysm was a bit far. Highly diluted N-butyl-2- cyanoacrylate (NBCA) is required due to the long distance in NBCA cases. However, highly diluted NBCA would impede the collaterals due to distant traveling, thereby causing ischemia. Conversely, coil placement would be too near. It has the possibility of recanalization through the anastomose.

## Conclusions

Ureteral branch artery embolization is rare but may be an effective and safe procedure.

## Data Availability

Not applicable.

## Abbreviations

TAE: Transcatheter arterial embolization
CT: Computed tomography
